# Does the pursuit of scientific excellence serve or hamper translational medical research: an historical perspective from hematological malignancies

**DOI:** 10.1038/s41408-022-00738-x

**Published:** 2022-10-07

**Authors:** Chris M. Bunce, Farhat L. Khanim, Mark T. Drayson

**Affiliations:** 1 School of Biosciences, University of Birmingham, Edgbaston, Birmingham, B15 2TT UK; 2 School of Biomedical Sciences, University of Birmingham, Edgbaston, Birmingham, B15 2TT UK; 3 Institute of Institute of Immunology and Immunotherapy, University of Birmingham, Edgbaston, Birmingham, B15 2TT UK

**Keywords:** Medical research, Therapeutics

## Abstract

Despite enormous global investment, translational medical research faces considerable challenges and patients, and their doctors are frequently frustrated by the apparent lack of research activity or progress. Understanding the factors that prevent innovative research discoveries from making it to clinical trials is a multifaceted problem. However, one question that must be addressed is whether the nature of current research activity and the factors that influence the conduct of pre-clinical research, permit, or hamper the timely progression of laboratory-based observations to proof of concept (PoC) clinical trials. Inherent in this question is to what extent a deep mechanistic understanding of a potential new therapy is required before commencing PoC studies, and whether patients are better served when mechanistic and clinical studies progress side by side rather than in a more linear fashion. Here we address these questions by revisiting the historical development of hugely impactful and paradigm-changing innovations in the treatment of hematological cancers. First, we compare the history and route to clinical PoC, of two molecularly-targeted therapies that are BCR:ABL inhibitors in chronic myeloid leukaemia and *all-trans* retinoic acid (ATRA) in acute promyelocytic leukaemia (APL). We then discuss the history of arsenic trioxide as additional APL therapy, and the repurposing of thalidomide as effective multiple myeloma therapy. These stories have surprising elements of commonality that demand debate about the modern-day hard and soft governance of medical research and whether these processes appropriately align the priorities of advancing scientific knowledge and the need of patients.

## The translation challenge facing medical research

There is little doubt that translational medical research faces significant challenges. Numerous articles continue to discuss the so-called ‘Valley of Death’ preventing innovative medical research discoveries from making it to clinical trials. Although a multifaceted problem, one component of this challenge is to establish the extent to which the mechanism of action of a potential new therapeutic intervention needs to be understood before it is appropriate to consider phase I/II trials.

This is a complex question, not least because the terminology and concepts surrounding these decisions are not always well defined in the minds of those that indirectly or directly influence the decisions. Therefore, to have this debate it is necessary to distinguish the phrases ‘*Mode of Action*’ and ‘*Mechanism of Action*’ which are both frequently abbreviated to MoA and in some contexts are unhelpfully used interchangeably. In the case of translational medical research, *Mode of Action* refers to the physical, or functional change caused by the action of a drug at the cellular level. In contrast a *Mechanism of Action* describes the action of the same drug at a molecular level. The other key term pertinent to this debate is *Proof of Concept* (PoC). This term refers to an investigation executed to test that a clinical concept is feasible, and most often refers to the execution of small-scale pilot clinical trials. PoC provides evidence that a drug is likely to be successful and, although often not published, PoC studies permit invested parties to make “Go/No-Go” decisions.

## Are the aspirations of academic biomedical research and translational medicine appropriately aligned?

An old proverb talks about big fleas having little fleas upon their backs to bite them, and little fleas have littler fleas and so *ad infinitum*. An increasing desire for deeper understanding of biochemical and molecular processes in health and disease inevitably creates yet more questions and more unknowns, fuelling the hunt for yet smaller ‘fleas’ that potentially delays or prevents potential progress for patients. Thus, a key aspect in this debate is what size of flea (depth of understanding), is appropriate to argue for clinical experimentation. In addressing this question, it is important to consider whether the perception of the appropriate ‘size of flea’ would be the same across the spectrum of non-clinical and clinical academics, other health professionals, researchers in industry, patients, or their carers? Also important to this debate is to recognise that a very significant proportion of translational medical research is funded by charities [1]. The primary objective of these organisations is patient outcome not necessarily complexity or depth of research knowledge. Thus, there is an additional moral imperative to limit unnecessary expenditure on laboratory studies where the evidence already exists to support early clinical investigation.

The academic pursuit of a definitive understanding of disease processes and corresponding *Mechanism of Action* of drugs proposed to target these processes has complex drivers. These include the career structure for research scientists that acts as a driver for ever more detailed experimentation to achieve publications in major journals that are seen as the necessary currency of job security, funding success and career progression. At the same time academic journals also operate in a competitive market and their success is linked to the ‘strength’ of the science they publish. In our experience this has led to a step change in the quantity and variety of data required to publish preclinical translational science in discipline-leading journals. The cumulative pressures of competitive funding and publishing of academic science, coupled with the inherent inquisitiveness of researchers, drives peer review processes to recommend/require very detailed work linked to deeper and deeper *Mechanism of Action* at the cost of alternative PoC studies that may more quickly and efficiently unlock clinical translation or alternatively demonstrate futility.

Therefore, the debate we would like to promote is whether the academic appetite for definitive *Mechanism of Action* is at odds with clinical need and whether in some situations this likely hampers rather than enhances medical progress.

## The view from blood cancers

**Fig. 1:**
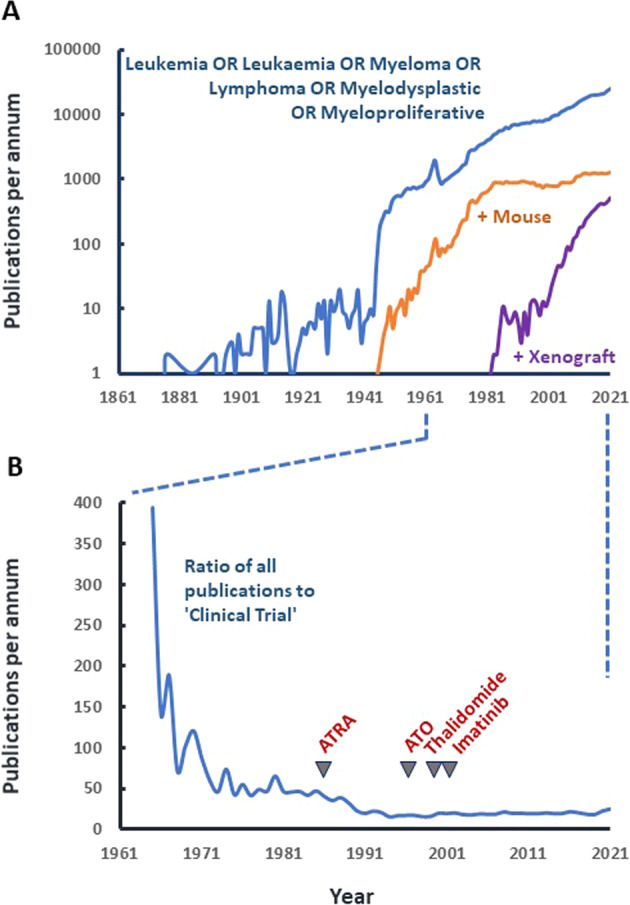
Numbers of Blood Cancer research articles and the proportion that include clinical trials. Data are from PubMed (03/05/2022). **A** Searches were for articles including the terms; (Leukemia OR Leukaemia OR Myeloma OR Lymphoma OR Myelodysplastic OR myeloproliferative) in either the title and/or legend (blue line), the same search with the inclusion of either (i) the term (mouse OR murine) either the title and/or legend (orange line) or (ii) the term (xenograft) either the title and/or legend (purple line). Publications are shown **B** Data from 1965–2021 showing the ratio of total publications retrieved using (Leukemia OR Leukaemia OR Myeloma OR Lymphoma OR Myelodysplastic OR myeloproliferative) in either the title and/or legend with the number retrieved using the same search phrase filtered by PuBMed ‘Clinical Trial’ function. Arrows indicate the timing of PoC studies for ATRA and ATO in APL, thalidomide in Myeloma and Imatinib in CML as discussed in the main text. The search strategies employed represent underestimates of total research activity as any studies currently in progress, abandoned unpublished are not included.

The post-war period has witnessed a huge and ongoing growth in research publications in the arena of blood cancers, including a significant proportion of papers using mouse models in their experiments (Fig. 1A). Interestingly since the 1980’s the proportion of these murine studies that have included xenograft experiments has risen sharply (Fig. 1A). In recent decades these growing publication rates have reached an equilibrium between studies that include and that do not include clinical trials (Fig. 1B). These observations may indicate that all is well in blood cancer research and that research activity is driving increasing translation toward appropriate clinical studies.

Indeed, history records that blood cancers have been exemplars in the paradigm for *Mechanism of Action* becoming the lynchpin to successful therapeutic breakthroughs. In this domain there are two historical and landmark molecularly well-understood therapies that have been transformative for patients; *all trans* retinoic acid (ATRA) therapy of acute promyelocytic leukaemia (APL) and BCR:ABL inhibitors in chronic myeloid leukaemia (CML). Whilst dramatic and hugely impactful, these important developments may have been a double-edged sword in the wider advancement of cancer and other therapies. Combined with the genomics explosion they have beguiled translational science that understanding the mutations present in individual cancers at a molecular level, will permit the design of magic bullet therapies with well-understood *Mechanism of Action*. Reciprocally they have placed the field in the headset that without such information it is too risky, either commercially or from a safety perspective, or unethical to advance to early exploratory clinical trials. Furthermore, the growing popularity of mouse models including xenograft models has made it difficult to advance to PoC studies without studies in mice. However, a closer inspection of the history of the above landmark achievements may inform the debate herein.

## Imatinib therapy for CML

The story of imatinib (Glivec) began with the recognition by Janet Rowley in 1973 that the CML-associated 22q- Philadelphia Chromosome (Ph^1^) [2] was in fact a translocation product with a reciprocal 9q+ chromosome [3] A decade later it became understood that most patients had a 22q-breakpoint within a defined 5.8 kb region that became known as the ‘breakpoint cluster region’ bcr and that the 9q component of the translocations involved the proto-oncogene c-abl. It was further identified that CML cells expressed a chimeric bcr:Aabl mRNA [4] and in 1986 David Baltimore and colleagues identified that a CML-associated 210-kilodalton phosphoprotein that cross reacted with v-abl-specific antisera was indeed the protein product of this fusion gene transcript [5]. Around the same time it was recognised that BCR:ABL chimeric proteins were hyperactive tyrosine kinases [6], that their intrinsic kinase activity was required for cell transformation [7], and that introduction of BCR:ABL alone produced leukaemias in mice [8, 9].

Tyrosine kinases universally use ATP as a phospho-donor and as a result it was widely assumed that targeting the ATP-binding pockets of kinases and kinase-oncoproteins, including BCR:ABL, would be non-selective and lead to widespread toxicities. However, Ciba Geigy (now Novartis) were amongst the first drug companies prepared to back the concept that ATP-mimetic Kinase inhibitors could be selective and generated a library of test compounds. The initial lead compound had weak activity against protein kinase-C and platelet derived growth factor-receptor (PDGF-R). Following chemical optimisation a series of more potent molecules were synthesised that exhibited dual inhibition of v-Abl and PDGF-r kinases and STI571 was adopted as the lead compound for pre-clinical development [10]. Although not specifically developed as targeted BCR:ABL therapy, laboratory studies by Brian Druker and others quickly demonstrated the activity of STI571 (later to be named Imatinib) against BCR:ABL positive cells in vitro and in mouse in vivo models [11–14]. In 1998 Druker and colleagues began a phase I dose escalation study that demonstrated that STI571 was well tolerated and had significant antileukemic activity in CML patients in whom treatment with interferon-alpha had failed [15]. Since then, imatinib and its second and third generation derivatives have transformed the therapeutic landscape for chronic phase CML, increasing ten-year survival rates from below 20% to more than 80% [16]. Many argue that with appropriate management, these drugs have returned chronic phase CML patients to normal or near normal life expectancy.

The story of imatinib is an example of how understanding the *Mechanism of Action* of an agent can successfully inform drug development. However, it was the demonstration of imatinib’s selective *Mode of Action* against CML cells in vitro and in vivo that opened the way to clinical trials and a subsequent paradigm shift in the treatment of this disease.

## ATRA therapy for APL

By the mid-1970s APL was recognised as a distinct entity amongst acute myeloid leukaemias (AML). In 1976 the French–American–British (FAB) Nomenclature Committee assigned the classification of M3, based on the distinct morphological characteristics of APL cells [17]. At the same time APL was recognised to be associated with abnormalities of chromosome 17 [18], that in 1977, were again identified by Janet Rowley and colleagues, as reciprocal translocations between the long arms of chromosomes 15 and 17, [19].

At the same time Leo Sachs was performing experiments in mouse myeloid leukaemias that indicated that the block in differentiation, in at least some leukaemia cells, could be reversed in vitro and in vivo [20–22]. In 1981 Theodore Breitman and colleagues also demonstrated in vitro differentiation of primary APL cells in the presence of ATRA [23] and a few years later Christine Chomienne demonstrated that the differentiating activity of ATRA was exclusive to primary APL cells and that other forms of AML did not respond. This work also identified that ATRA was particularly potent at differentiating APL cells as the entire cell population was differentiated into neutrophil-like cells in 7 days of culture [24] therefore establishing the *Mode of Action* of ATRA in APL. At the time ATRA was not manufactured in Europe or the USA. Hoffman-Laroche preferred to produce 13-cis retinoid acid as proven less toxic. However, the French group treated two patients who could not benefit from standard chemotherapy with 13-cis retinoic acid and two further 3^rd^ relapse APL patients with ATRA provided by Prof Wang Zhen Yi from Shanghai, where ATRA was approved for the treatment of skin diseases such as psoriasis and acne. Neither patient treated with 13-*cis* retinoic acid demonstrated improved haemopoiesis, though the APL cells were induced to differentiate in vitro with high concentrations. In contrast in the ATRA treated patients, neutrophil counts and haemoglobin levels returned to normal within 18 days and 90 days respectively and both patients demonstrated clinically significant improvements in platelet counts. Normal bone marrow maturation and karyotype were observed in both patients within 62 days treatment [25]. Similarly the Chinese group reported on 6 newly diagnosed and refractory APL patients treated with ATRA who all entered into complete remission (CR) [26] and a subsequent series of 24 patients in which all but one achieved CR when given ATRA alone and one that failed to respond to ATRA alone, but achieved CR upon the addition of cytosine arabinoside (Ara-C) [27]. These early studies provided unquestionable PoC for ATRA in APL and within a few years, multicenter randomized trials had demonstrated that, although the CR rates of APL patients treated with daunorubicin-Ara-C chemotherapy (CT) alone were not significantly different from those also treated with ATRA, the long-term outcome of patients treated with ATRA was better than that for patients with CT alone [28, 29]. To this day ATRA remains a cornerstone of APL treatment.

In stark contrast to the imatinib in CML story, *Mechanism of Action* of ATRA in APL was completely unknown when it became accepted as the way forward for these patients. The first patient to be treated with ATRA was treated in Shanghai’s Children’s Hospital in 1985 (for an account see [30]). However, RARα was not identified as the nuclear receptor for ATRA until two years later in the laboratories of Ron Evans and Pierre Chambon [31, 32]. A year later the gene for RARα was mapped to chromosome 17 q21 [33] and a number of studies ensued that confirmed RARα disruption in APL t15:17 translocations. In 1990 Hugues de Thé, Christine Chomienne and others detected fusion mRNA transcripts in APL cells, quickly followed a year later by reports from the laboratories of Hugues de Thé and Ron Evans, that the fusion transcripts encode a fusion protein between RARα and a novel protein which became known as PML. Furthermore, the fusion protein was shown to function as a dysregulated retinoic acid receptor [34, 35].

**Fig. 2:**
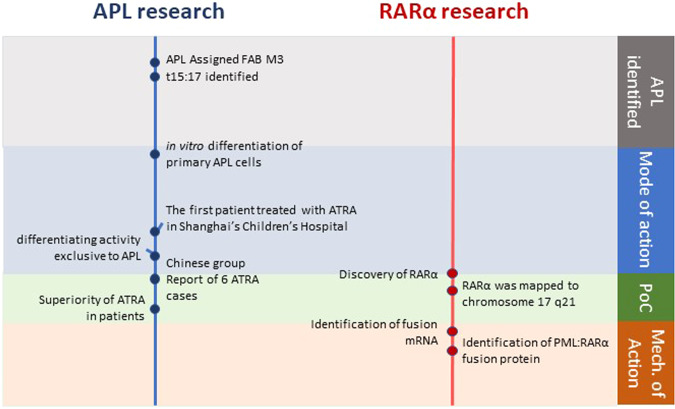
The historical intimacy of ATRA being rolled out as therapy in APL patients, the discovery of RARα and the subsequent discovery of PML:RAR. Schematic representation of the of the separate timelines of the discovery of ATRA as therapy for APL (including the (i) identification of APL as a distinct morphological and karyotypic entity in AML (ii) the identification of the *Mode of Action* of ATRA in APL and the ensuing clinical PoC studies) and the independent discovery of RARα as the receptor for ATRA that permitted the post PoC identification of the *Mechanism of Action* of ATRA as APL therapy.

Thus, identifying that the PML:RARα fusion protein provided the basis of *Mechanism of Action* for ATRA in APL was reported six years after the first PoC patient was treated and post-dating the first reports of *Mode of Action*-informed larger clinical trials by 2-3 years (Fig. 2). Nevertheless, over time, the retrospective historical intimacy of ATRA being rolled out as therapy in patients, the discovery of RARα and the subsequent discovery of PML:RAR (Fig. 2) have skewed popular understanding toward believing ATRA was a ‘designer’ PML:RAR-targeted therapy in the same way that Imatinib was selected to target BCR:ABL. In truth the ATRA-APL story shows a different route to success where clinical potential and understanding of *Mechanism of Action* have developed in parallel rather than following a more linear ‘*Mechanism of Action first, trial later*’ approach. Looking back on the development of ATRA therapy for APL it was again the selective differentiation and killing of APL cells that was seen to provide adequate *Mode of Action* that was quickly followed by PoC in the treatment of small numbers of patients in both China and France.

## Arsenic trioxide (ATO) in APL

The identification of the PML:RARα fusion protein spawned a plethora of laboratory studies over the ensuing years with ever increasingly deeper understanding of the molecular mechanisms of RARα function and its dysregulation underpinning APL. This largely academically driven research was the focus of substantial funding investments and research time around the world and the subject of a large volume of published studies. However, importantly for the debate herein, this research and its associated enhanced understanding of *Mechanism of Action* was not the source of the next great step in APL treatment. This came from a different and unexpected direction and was led by clinical rather than laboratory investigation.

The late 1990’s saw a number of studies published by groups in China demonstrating in vitro and in vivo clinical activity of arsenic trioxide (ATO) against APL. These publications were the culmination of PoC clinical studies undertaken in China from the 1970’s onwards. Again, cell-based *Mode of Action* studies rather than molecular-based *Mechansim of Action* studies were perceived to be appropriate to advance to clinical PoC studies. In 1997 Zhi-Xiang Shen et al. reported clinical CRs in 9 of 10 patients treated with ATO as monotherapy after relapse following ATRA, and CRs in another 5 ATRA-relapse patients rescued with ATO in combination with chemotherapy or ATRA [36]. Since these early studies provided the PoC for ATO in APL, hundreds of laboratory-based studies have followed seeking molecular clarification of the molecular *Mechanism of Action* of ATO in APL. However, as was the case with ATRA this understanding grew only in the wake of the evidence of its efficacy in patients and has added very little to the way patients are treated.

In contrast, since the seminal papers of the 1990’s establishing both ATRA and ATO as therapy in APL, treatment optimisation combining these agents has grown apace leading to the development of a relatively low toxicity route to a cure for most APL patients. Laboratory science has had its impact on this journey for example informing patient stratification and measurement of residual disease, but the major strides have been made in meticulous peer-reviewed approaches to clinical trial design, execution, and analysis. In addition, it is important to note that ATRA, ATO, and combinations thereof, were proven effective in patients without extensive experimentation in animal models. This argues against the growing paradigm in the modern-day academic setting of translational blood cancer research that mouse studies are a prerequisite for early PoC intervention studies.

## Thalidomide in myeloma

Vincent Devita and Edward Chu’s excellent article *A History of Cancer Chemotherapy* explains that in the 1960s there was extensive scepticism surrounding the promise of small molecule chemotherapy in cancer [37]. It is therefore interesting to note that the story of Thalidomide in Multiple Myeloma (MM) has its origins in this time. In 1963 the United States Eastern Cooperative Group in Solid Tumor Chemotherapy began a study of the tolerability and efficacy of Thalidomide in cancer. The rationale for the trial was relatively weak but was based on the excellent safety profile of the drug when administered outside of pregnancy and the then urgent need for any novel cancer therapeutics. Twenty-one patients with a spectrum of cancers were treated including 2 MM patients. One MM patient died with progressive disease (PD) after 8 weeks of therapy whilst the other displayed ‘subjective improvement’ before stopping therapy after 16 weeks with PD [38].

In 1997 Bart Barlogie and colleagues began a study that revisited Thalidomide as therapy for refractory MM patients for whom there were very few therapeutic options at that time. The rationale for this trial was that thalidomide had been recently identified as anti-angiogenic [39, 40] and that increased bone marrow vascularity had been demonstrated as a marker of poor prognosis in MM [41, 42]. This inferred a potential *Mode of Action* against MM that however had not been tested directly in a MM setting before undertaking PoC studies.

The study first administered Thalidomide to five patients with end-stage MM through a compassionate-use protocol [43]. One patient with more than 95% myeloma cells in the bone marrow who had not responded to two cycles of high-dose chemotherapy, had near complete remission within 3 months of beginning Thalidomide therapy. Based on this PoC the group proceeded to a phase II trial of Thalidomide in advanced and refractory MM patients [43].

At the time of commencing this trial, no in vitro, or in vivo studies of the effect of thalidomide against MM had been undertaken and a potential *Mode of Action* could only be inferred. However, 27 of 84 (32%) treated patients had a fall in serum or urine paraprotein (a measure of MM tumour burden) of 25% or more, with 8 patients having a fall of 90% or more [43]. After 12 months follow-up, overall survival in the trial was a remarkable 58 ± 5% [43]. Interestingly, the microvascular density of bone marrow did not change significantly in patients with a response [43].

Despite having uncertain *Mode* or *Mechanism of Action* this ground-breaking, carefully designed, compassionate and ethically robust two-stage clinical trial fundamentally changed the future landscape for MM patients. As in the case of ATRA and ATO in APL, *Mechanism of Action* studies have run in parallel with clinical studies and Thalidomide and its derivatives Lenalidomide, Pomalidomide are now considered immunomodulatory drugs (IMiDs) with complex *Mechanisms of Action* that are still being unravelled to this day.

## Concluding remarks

Together the stories of Imatinib, ATRA/ATO and Thalidomide, describe the remarkable potential of translational medical research. What sets the ATRA/ATO and Thalidomide stories apart from that of Imatinib is that clinical trials were commenced with little molecular understanding of *Mechanism of Action*. These hugely successful historical precedents must challenge how in the modern day we set the parameters for appropriate progression to clinical PoC interventions.

What also sets these stories apart is that clinical grade ATRA, ATO and thalidomide were available for use in APL and MM whereas drugs that targeted BCR:ABL had to be developed anew. Combined these iconic stories illustrate the value in both new drug discovery and drug repurposing strategies. In this regard, it is important to consider whether ATRA/ATO and thalidomide followed a quicker path to PoC studies in the absence of *Mechanism of Action*, merely because they were available to haematologists and could be tested outside of the drug discovery pipeline of a particular pharmaceutical company. We would argue that this viewpoint is too simplistic. The time to PoC studies with imatinib, once it had been identified in screens as a selective BCR:ABL inhibitor and *Mode of Action* had been demonstrated by selective killing of CML cells in vitro and in vivo models, was also short and comparable to the timeframe of *Mode of Action* and PoC transitions in the case of ATRA and ATO in APL. The argument is more subtle. What these important exemplars show is that practice was changed by early PoC studies undertaken when patients and their doctors deemed the risk-benefit criteria to be appropriate.

The question remains would these stories be the same today under the current climate of perceived need for detailed mechanistic research? All the above stories are united by being driven by passionate, talented, and motivated clinical researchers determined to improve the outcomes for their patients. The stories illustrate that the degree of understanding of *Mode of Action* that is acceptable in the clinic to both patients and their doctors is not a constant but contextual. Reciprocally therefore, there is a danger that science and academia, in their pursuit of excellence, may delay or indeed prevent potential medical discoveries from ever reaching the clinic.

Of course, it would be obtuse not to acknowledge that the immediacy and severity of the clinical need in cancer patients for whom no other effective therapy is available, alters the appetite of doctors and patients to undertake PoC studies and clinical trials. This means that what is ethical in this scenario may not be deemed ethical in other less urgent medical conditions. Be that as it may, we would argue that this ethical debate should take place in a wider setting than that of academic science research which may unwittingly be wrongfully setting the agenda in the pursuit of premature deeper molecular understanding.

In drug development both patient safety and patient benefit are of paramount importance. We do not advocate progression to clinical trials with newly developed drugs without preclinical in vivo toxicity testing in mice or larger animals. However, from the point of view of the reduction, refinement and replacement of animals in research, the historical journeys of Imatinib, ATRA, ATO and Thalidomide, in shifting patient outcomes, required minimal or no in vivo experiments prior to establishing paradigm-shifting PoC clinical trials. This is at odds with the current norm (at least in academic blood cancer research) that mechanism/mode of action evidence in mouse models is a prerequisite of translational research. This again questions whether academia has the appropriate understanding of the routes to translation and whether ‘academic standards’, particularly those of non-clinical scientists, drive more excessive preclinical research than required; in turn driving up the time and expense of this type of research which is so often funded by charities and other forms of public spending.

Although we focus on the impact academic drive has on the preclinical research pathway it is important to note that other forces are at play. Not least is the evolving ethical and regulatory approval landscape that make it increasing difficult to proceed with PoC studies without very extensive preclinical data. It is also important to consider the impact of the pharmaceutical industry on the nature of the preclinical research pathways and what their priorities are in terms of routes to drug development. It is arguably easier for trials of re-purposed drugs to be clinician-led and freer from influence from Pharma. However, it is also possible to argue that Pharma-led drug development would benefit from the earliest possible transition to exploratory trials and allow them to make better go/no-go decisions on compounds in their development pathways. Even when these trials fail to meet the criteria of commercial or clinical success it is imperative that the outcomes are published. For example, they may provide proof of safety for future trials of a drug in other settings as illustrated in the case of thalidomide.

## Data Availability

Data sharing not applicable to this article as no datasets were generated or analysed during the current study.

## References

[1] Griffiths C, Mitchell M, Burnand A (2020). More support needed for UK charity-funded medical research. Lancet.

[2] Nowell PC (1962). The minute chromosome (Phl) in chronic granulocytic leukemia. Blut.

[3] Rowley JD (1973). Letter: A new consistent chromosomal abnormality in chronic myelogenous leukaemia identified by quinacrine fluorescence and Giemsa staining. Nature.

[4] Heisterkamp N, Stam K, Groffen J, de Klein A, Grosveld G (1985). Structural organization of the bcr gene and its role in the Ph’ translocation. Nature.

[5] Ben-Neriah Y, Daley GQ, Mes-Masson AM, Witte ON, Baltimore D (1986). The chronic myelogenous leukemia-specific P210 protein is the product of the bcr/abl hybrid gene. Science.

[6] Davis RL, Konopka JB, Witte ON (1985). Activation of the c-abl oncogene by viral transduction or chromosomal translocation generates altered c-abl proteins with similar in vitro kinase properties. Mol Cell Biol.

[7] Lugo TG, Pendergast AM, Muller AJ, Witte ON (1990). Tyrosine kinase activity and transformation potency of bcr-abl oncogene products. Science.

[8] Daley GQ, Van Etten RA, Baltimore D (1990). Induction of chronic myelogenous leukemia in mice by the P210bcr/abl gene of the Philadelphia chromosome. Science.

[9] Kelliher MA, McLaughlin J, Witte ON, Rosenberg N (1990). Induction of a chronic myelogenous leukemia-like syndrome in mice with v-abl and BCR/ABL. Proc Natl Acad Sci USA.

[10] Mauro MJ, Druker BJ (2001). STI571: a gene product-targeted therapy for leukemia. Curr Oncol Rep.

[11] Druker BJ, Tamura S, Buchdunger E, Ohno S, Segal GM, Fanning S (1996). Effects of a selective inhibitor of the Abl tyrosine kinase on the growth of Bcr-Abl positive cells. Nat Med.

[12] Deininger MW, Goldman JM, Lydon N, Melo JV (1997). The tyrosine kinase inhibitor CGP57148B selectively inhibits the growth of BCR-ABL-positive cells. Blood.

[13] Gambacorti-Passerini C, le Coutre P, Mologni L, Fanelli M, Bertazzoli C, Marchesi E (1997). Inhibition of the ABL kinase activity blocks the proliferation of BCR/ABL+ leukemic cells and induces apoptosis. Blood Cells Mol Dis.

[14] le Coutre P, Mologni L, Cleris L, Marchesi E, Buchdunger E, Giardini R (1999). In vivo eradication of human BCR/ABL-positive leukemia cells with an ABL kinase inhibitor. J Natl Cancer Inst.

[15] Druker BJ, Talpaz M, Resta DJ, Peng B, Buchdunger E, Ford JM (2001). Efficacy and safety of a specific inhibitor of the BCR-ABL tyrosine kinase in chronic myeloid leukemia. N. Engl J Med.

[16] Hochhaus A, Larson RA, Guilhot F, Radich JP, Branford S, Hughes TP (2017). Long-Term Outcomes of Imatinib Treatment for Chronic Myeloid Leukemia. N. Engl J Med.

[17] Bennett JM, Catovsky D, Daniel MT, Flandrin G, Galton DA, Gralnick HR (1976). Proposals for the classification of the acute leukaemias. French-American-British (FAB) co-operative group. Br J Haematol.

[18] Golomb HM, Rowley J, Vardiman J, Baron J, Locker G, Krasnow S (1976). Partial deletion of long arm of chromosome 17: a specific abnormality in acute promyelocytic leukemia?. Arch Intern Med.

[19] Rowley JD, Golomb HM, Dougherty C (1977). 15/17 translocation, a consistent chromosomal change in acute promyelocytic leukaemia. Lancet.

[20] Paran M, Sachs L, Barak Y, Resnitzky P (1970). In vitro induction of granulocyte differentiation in hematopoietic cells from leukemic and non-leukemic patients. Proc Natl Acad Sci USA.

[21] Fibach E, Hayashi M, Sachs L (1973). Control of normal differentiation of myeloid leukemic cells to macrophages and granulocytes. Proc Natl Acad Sci USA.

[22] Gootwine E, Webb CG, Sachs L (1982). Participation of myeloid leukaemic cells injected into embryos in haematopoietic differentiation in adult mice. Nature.

[23] Breitman TR, Collins SJ, Keene BR (1981). Terminal differentiation of human promyelocytic leukemic cells in primary culture in response to retinoic acid. Blood.

[24] Chomienne C, Balitrand N, Degos L, Abita JP (1986). 1-B-D arabinofuranosyl cytosine and all-trans retinoic acid in combination accelerates and increases monocyte differentiation of myeloid leukemic cells. Leuk Res.

[25] Chomienne C, Ballerini P, Balitrand N, Amar M, Bernard JF, Boivin P (1989). Retinoic acid therapy for promyelocytic leukaemia. Lancet.

[26] Huang ME, Ye YC, Chen SR, Zhao JC, Gu LJ, Cai JR (1987). All-trans retinoic acid with or without low dose cytosine arabinoside in acute promyelocytic leukemia. Rep. 6 cases Chin Med J (Engl).

[27] Huang ME, Ye YC, Chen SR, Chai JR, Lu JX, Zhoa L (1988). Use of all-trans retinoic acid in the treatment of acute promyelocytic leukemia. Blood.

[28] Fenaux P, Le Deley MC, Castaigne S, Archimbaud E, Chomienne C, Link H (1993). Effect of all transretinoic acid in newly diagnosed acute promyelocytic leukemia. Results of a multicenter randomized trial. European APL 91 Group. Blood.

[29] Tallman MS, Andersen JW, Schiffer CA, Appelbaum FR, Feusner JH, Woods WG (2002). All-trans retinoic acid in acute promyelocytic leukemia: long-term outcome and prognostic factor analysis from the North American Intergroup protocol. Blood.

[30] Wang ZY, Chen Z (2008). Acute promyelocytic leukemia: from highly fatal to highly curable. Blood.

[31] Giguere V, Ong ES, Segui P, Evans RM (1987). Identification of a receptor for the morphogen retinoic acid. Nature.

[32] Petkovich M, Brand NJ, Krust A, Chambon P (1987). A human retinoic acid receptor which belongs to the family of nuclear receptors. Nature.

[33] Mattei MG, Petkovich M, Mattei JF, Brand N, Chambon P (1988). Mapping of the human retinoic acid receptor to the q21 band of chromosome 17. Hum Genet.

[34] Kakizuka A, Miller WH, Umesono K, Warrell RP, Frankel SR, Murty VV (1991). Chromosomal translocation t(15;17) in human acute promyelocytic leukemia fuses RAR alpha with a novel putative transcription factor, PML. Cell.

[35] de The H, Lavau C, Marchio A, Chomienne C, Degos L, Dejean A (1991). The PML-RAR alpha fusion mRNA generated by the t(15;17) translocation in acute promyelocytic leukemia encodes a functionally altered RAR. Cell.

[36] Shen ZX, Chen GQ, Ni JH, Li XS, Xiong SM, Qiu QY (1997). Use of arsenic trioxide (As2O3) in the treatment of acute promyelocytic leukemia (APL): II. Clinical efficacy and pharmacokinetics in relapsed patients. Blood.

[37] DeVita VT, Chu E (2008). A history of cancer chemotherapy. Cancer Res.

[38] Olson KB, Hall TC, Horton J, Khung CL, Hosley HF (1965). Thalidomide (N-Phthaloylglutamimide) in the Treatment of Advanced Cancer. Clin Pharm Ther.

[39] D’Amato RJ, Loughnan MS, Flynn E, Folkman J (1994). Thalidomide is an inhibitor of angiogenesis. Proc Natl Acad Sci USA.

[40] Kenyon BM, Browne F, D’Amato RJ (1997). Effects of thalidomide and related metabolites in a mouse corneal model of neovascularization. Exp Eye Res.

[41] Vacca A, Ribatti D, Roncali L, Ranieri G, Serio G, Silvestris F (1994). Bone marrow angiogenesis and progression in multiple myeloma. Br J Haematol.

[42] Vacca A, Di Loreto M, Ribatti D, Di Stefano R, Gadaleta-Caldarola G, Iodice G (1995). Bone marrow of patients with active multiple myeloma: angiogenesis and plasma cell adhesion molecules LFA-1, VLA-4, LAM-1, and CD44. Am J Hematol.

[43] Singhal S, Mehta J, Desikan R, Ayers D, Roberson P, Eddlemon P (1999). Antitumor activity of thalidomide in refractory multiple myeloma. N. Engl J Med.

